# A higher posterior tibial slope and tunnel malposition do not predispose to non‐traumatic anterior cruciate ligament failure

**DOI:** 10.1002/jeo2.70699

**Published:** 2026-04-29

**Authors:** Lorenz Fritsch, Lukas Willinger, Romed P. Vieieder, Luca Bausch, Julian Mehl, Svenja Höger, Sebastian Siebenlist, Armin Runer

**Affiliations:** ^1^ Department of Sports Orthopaedics TUM University Hospital Munich Germany

**Keywords:** ACL, ACLR, ACL‐reconstruction, ACL‐revision, meniscus

## Abstract

**Purpose:**

A higher posterior tibial slope (PTS) and malpositioning of bone tunnels are known risk factors for anterior cruciate ligament reconstruction (ACL reconstruction; ACLR) failure. However, it remains unclear whether these factors account for the mechanism of failure, whether traumatic or non‐traumatic. The purpose of this study was to analyze whether the ACL failure mechanism correlates with a higher PTS or non‐anatomical bone tunnels. It was hypothesized that a higher PTS and bone tunnel malposition are associated with non‐traumatic ACLR failure.

**Methods:**

In this retrospective study, all ACLR failures between 2015 and 2023 treated surgically at a single institution were included. The following factors were evaluated: sex, age at the time of revision surgery, PTS, anatomical versus non‐anatomical tunnel placement, concomitant pathologies and fixation techniques. PTS was measured using the Dejour technique. Tibial and femoral tunnels were analyzed according to the method described by Stäubli and Rauschning, and Bernard et al., respectively. A logistic regression analysis was performed to evaluate the effect of each factor on the mechanism of graft failure (traumatic vs. non‐traumatic).

**Results:**

Data from 143 of 144 available patients (99.3%) were included. There was no statistically significant difference in patient demographics between the two groups (*p* > 0.05). There was no statistically significant association between the PTS or tunnel malposition and non‐traumatic ACLR failure (*p* > 0.05). However, medial meniscus injuries were significantly more frequent in the non‐traumatic group (*n* = 53 [54.1%] vs. *n* = 16 [35.6%]; *p* = 0.034). The logistic regression showed no significant impact of any studied factor (*p* > 0.05) on the mechanism of ACLR failure.

**Conclusion:**

Patients in our cohort with non‐traumatic ACLR failure did not demonstrate a higher PTS or a higher incidence of non‐anatomically positioned tibial or femoral tunnels compared with patients experiencing traumatic retears. However, non‐traumatic cases were associated with a higher prevalence of medial meniscus tears.

**Level of Evidence:**

Level III, retrospective comparative study.

**Abbreviations**
ACL: anterior cruciate ligament
ACLR: anterior cruciate ligament reconstruction
BMI: body mass index
IQR: interquartile range
PTS: posterior tibial slope

## INTRODUCTION

Revision anterior cruciate ligament (ACL) reconstruction (ACLR) is demanding, shows unfavorable clinical outcomes and higher failure rates compared to primary ACLR [10, 12, 18, 27, 33]. Previous studies have identified multiple factors contributing to ACLR failure with particular emphasis on biological causes and technical errors, including malpositioning of bone tunnels and the use of allografts [6, 7, 9, 18, 26]. Moreover, incorrect tunnel placement has been described as a major risk factor for non‐traumatic graft failure [5, 22].

A higher posterior tibial slope (PTS) > 12° [1, 13, 21, 30, 31, 32], concomitant meniscal and collateral ligament injuries, increased anterolateral rotatory knee laxity [4, 20], coronal malalignment [24] and increased medial laxity [25] have been identified as factors influencing the risk of ACLR failure. While there is certainly a linear correlation between increasing PTS and a higher rerupture rate, a PTS > 12° is associated with an increased failure rate [13, 21]. Additionally, a recent work revealed that non‐traumatic failures tend to obtain a significantly increased PTS [22].

The aim of this study was to evaluate the impact of an elevated PTS and tunnel malposition on the traumatic versus non‐traumatic onset of ACLR failure. It was hypothesized that a higher PTS and malpositioned tunnels significantly contribute to the occurrence of a non‐traumatic ACLR failure.

## MATERIAL AND METHODS

The present retrospective study has been approved by an Institutional Review Board (2022‐527‐S‐NP). Patients who underwent revision ACLR between 2015 and 2023 at a single institution were included. Moreover, 124 (88.7%) of the included patients received their primary ACLR at an external institution. Demographic information, details regarding the injury mechanism, concomitant injuries and procedures were retrieved from the institutional electronic medical records. In cases where the injury mechanism was not clearly documented, patients were contacted by telephone to obtain precise information regarding the circumstances of the injury. The etiology of the re‐injury was classified into traumatic and non‐traumatic, and patients were divided into two study groups accordingly. ACLR failure was classified as ‘traumatic’ if it occurred in direct association with a distinct injury event, typically involving high‐energy or abnormal knee loading (e.g., during sports participation, falls or accidents). When no identifiable traumatic incident led to the ACLR failure, it was classified as ‘non‐traumatic’. Exclusion criteria included cases in which the onset of injury was not clearly defined or patients were unavailable for follow‐up.

### Radiological measurement

**Figure 1:**
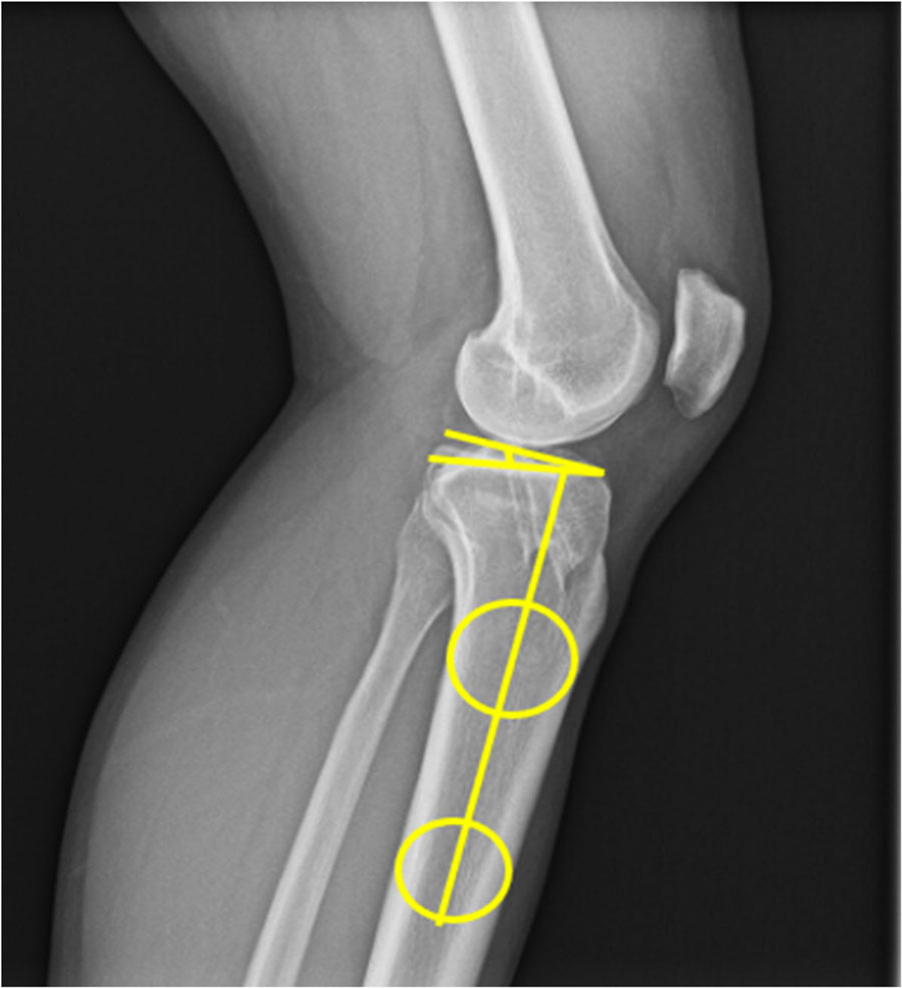
Assessment of the posterior tibial slope (PTS) on a strict lateral radiograph using the centers of two circles placed in the tibial shaft to determine the proximal tibial axis. The PTS was then measured as the angle between an orthogonal line relative to the proximal axis and a tangent of the medial tibial plateau [8].

The PTS was measured postoperatively on strict lateral x‐rays according to a previously described and established method [8]. Two circles were placed within the tibial shaft, tangent to the anterior and posterior cortex; the superior circle was positioned just below the tibial tubercle and the inferior circle about 10 cm distal to the superior one. The proximal anatomical tibial axis was defined by the line passing through the center of both circles. A horizontal reference line was then placed perpendicular to the proximal anatomical axis. Subsequently, a tangential line was drawn across the central portion of the lateral and the medial tibial plateau, representing the dorsal tibial inclination. The PTS was defined as the angle between the horizontal reference line and the tangential line (Figure 1).

**Figure 2:**
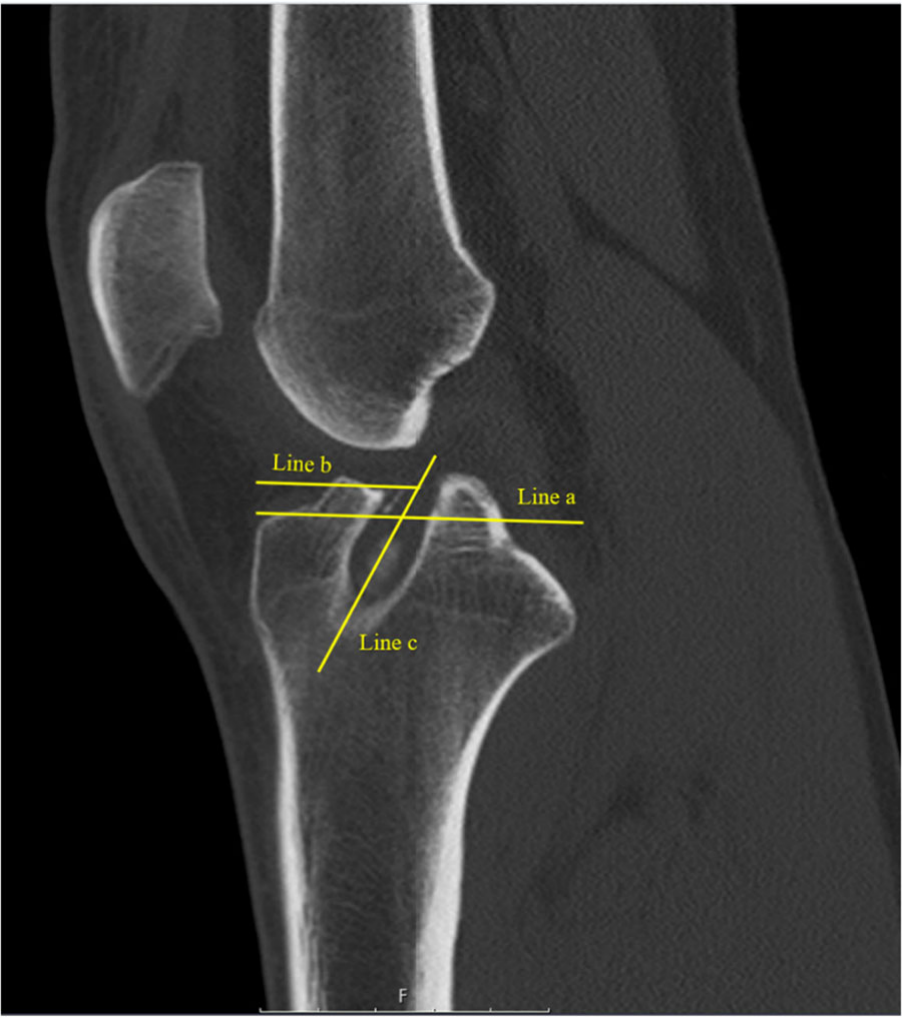
Determination of the location of the tibial tunnel on sagittal computed tomography [23]. (Line a) maximum anterior‐posterior diameter of the tibia (= 100%). (Line b) distance to the center of the tibial tunnel. (Line c) center of the tibial tunnel.

The tibial and femoral tunnel positions were assessed on computed tomography (CT) scans according to a previously established method [16, 17]. To evaluate the tibial tunnel, the maximum anteroposterior diameter of the tibial plateau was measured. Subsequently, the anteroposterior diameter of the tibial plateau to the center of the tibial tunnel was determined [23]. The anatomical center of the tunnel has been described to be located at approximately 43% of its maximum anteroposterior diameter, with an acceptable variation of 9% [23]. In the present study, a tibial tunnel was considered anatomic when the tunnel center was located within 43% ± 5% of the maximum anteroposterior tibial plateau diameter and non‐anatomic outside of this range (Figure 2).

**Figure 3:**
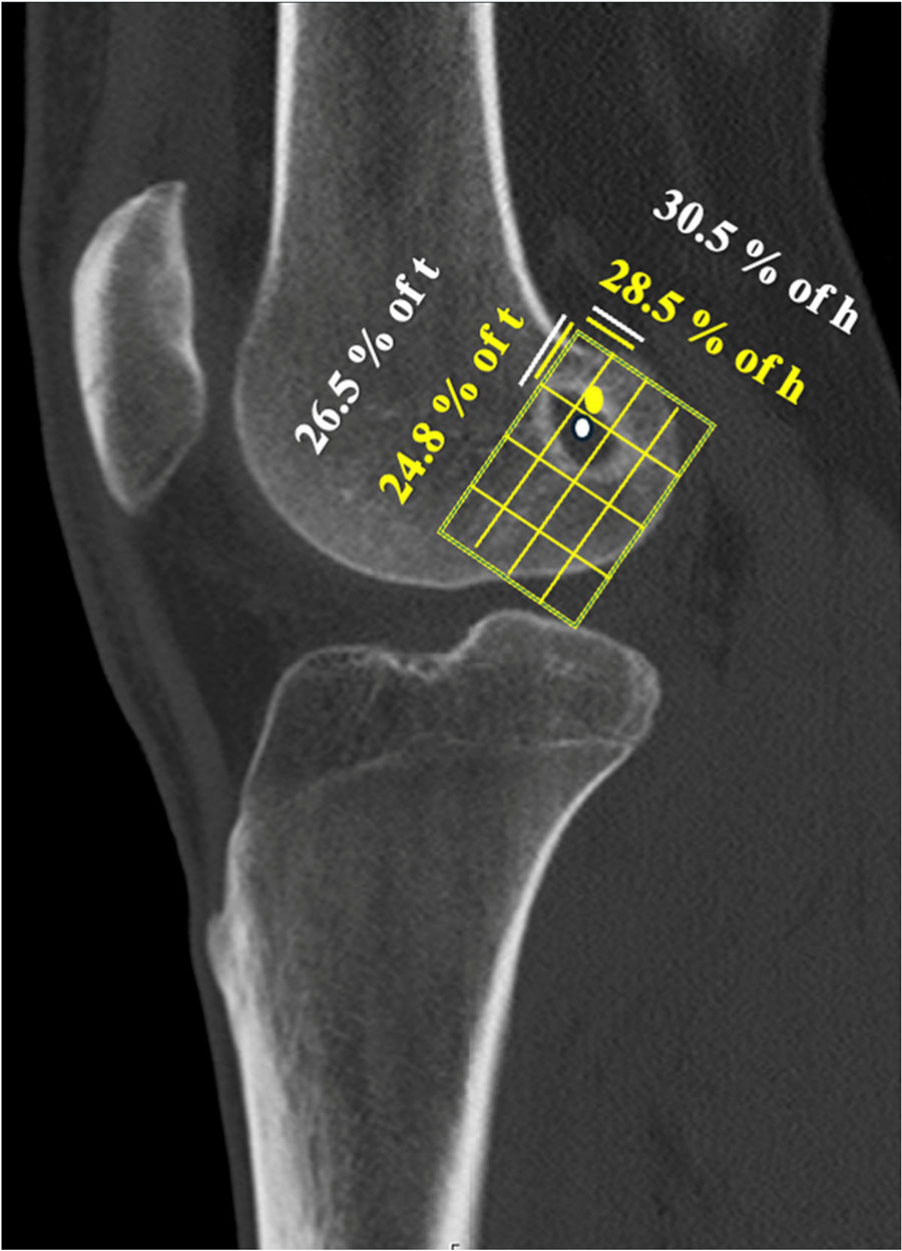
The anatomic center of the femoral anterior cruciate ligament (ACL) footprint is indicated by a yellow dot, located at 24.8% along the Blumensaat line (*t*‐axis) and 28.5% along the perpendicular head (*h*‐axis) [3]. The white dot represents the center of the femoral tunnel of this specific patient. The white *t*‐ and *h*‐coordinates define the tunnel's position relative to the anatomic center. In the displayed image, the tunnel center deviated by less than 10% from the anatomic origin, and therefore, it was classified as anatomic.

Evaluation of the femoral tunnel was conducted using a method previously established for lateral radiographs and subsequently validated for CT [2, 3, 16, 17]. Multiplanar reconstructions were generated to obtain a true sagittal view of the lateral femoral condyle. A reference rectangle was then superimposed on the lateral wall of the intercondylar notch: the superior border was aligned with the Blumensaat line, the inferior border with the most distal point of the lateral condyle and the anterior and posterior borders with respect to the cortical margins. The length of the Blumensaat line defined the proximal–distal axis (*t*), while the perpendicular height of the rectangle defined the anterior–posterior axis (*h*) (Figure 3). The center of the femoral tunnel aperture was identified on CT and expressed as a percentage of both axes (*t* and *h*). The center of the femoral tunnel aperture is considered anatomic at the intersection of 24.8% along line *t* and 28.5% along line *h*. A deviation of ±2.5% from this reference point is generally accepted as within the anatomic range [2, 3]. In the present study, a broader tolerance of ±10% was applied for the final analysis when defining a cutoff for a non‐anatomic femoral tunnel position. The originally defined cut‐off of 2.5% would have created a range that was too narrow for tunnels lying outside this interval but still exhibiting an essentially good anatomical position.

### Interrater reliability

Interrater reliability was assessed for both the measurement of PTS and the definition of femoral tunnel position. Two independent raters carried out the measurements.

### Statistical analysis

Descriptive statistics were conducted using SPSS software version 26.0 (IBM‐SPSS). Continuous variables were reported as mean ± standard deviation or median with interquartile range (IQR), and categorical variables were presented as numbers and percentages, depending on data distribution assessed by the Kolmogorov–Smirnov test. Logistic regression analysis, with the injury mechanism of ACLR failure (traumatic versus non‐traumatic) as the dependent variable, was performed to evaluate the influence of sex, age, number of previous ACL surgeries, PTS, tibial and femoral tunnel position as well as the fixation technique of the graft. Statistical significance was considered at *p* < 0.05.

## RESULTS

### Demographics

A total of 143 (99.3%) of 144 eligible patients were included in the final analysis. One patient (0.7%) was lost to follow‐up. Demographic characteristics, including the tunnel position and the mean PTS of the patient cohort, are provided in Table 1. No statistically significant differences were detected in the patients' demographic factors (*p* > 0.05).

**Table 1:** Demographics of patients with ACL graft failure according to their mechanism of failure.

	Traumatic	Non‐traumatic	*p* value
Number of patients	45 (31.5%)	98 (68.5%)	
Sex (*n*)			
Male	30 (67%)	74 (75.5%)	0.31
Female	15 (33%)	24 (24.5%)	0.31
Age at latest revision surgery (years)	27.7 ± 8.8	29.9 ± 8.9	0.92
BMI (kg/cm^2^)	26.9 ± 5.6	25.5 ± 4.6	0.13
Revision surgery			
1 surgery	39 (87%)	77 (78.6%)	0.25
>1 surgery	6 (13%)	21 (21.4%)	0.13
PTS (°)	9.8 ± 3.5	10.5 ± 3.9	0.37
PTS < 12°	29 (64.4)	65 (66.3)	0.82
PTS > 12°	16 (35.5)	33 (33.7)	0.82
Non‐anatomic tunnel n (%)			
Femoral, *n* (%)	30 (66.6)	73 (74.4)	0.65
Tibial, *n* (%)	27 (60.0)	44 (44.9)	0.1
Distance to coordinate, *h* (%)	17.8 ± 10.1	17.9 ± 13.4	0.97
Distance to coordinate, *t* (%)	18.4 ± 4.2	17.1 ± 7.4	0.22
Tibial tunnel position (%)	0.45 ± 0.06	0.44 ± 0.07	0.62

*Note*: Normally distributed continuous variables are shown as mean ± standard deviation. Absolute values and their distribution are reported as a number (%). The tibial tunnel position is expressed as a percentage of the maximum ap distance of the tibial plateau.

Abbreviations: ACL, anterior cruciate ligament; BMI, body mass index; PTS, posterior tibial slope.

### Surgical results

**Figure 4:**
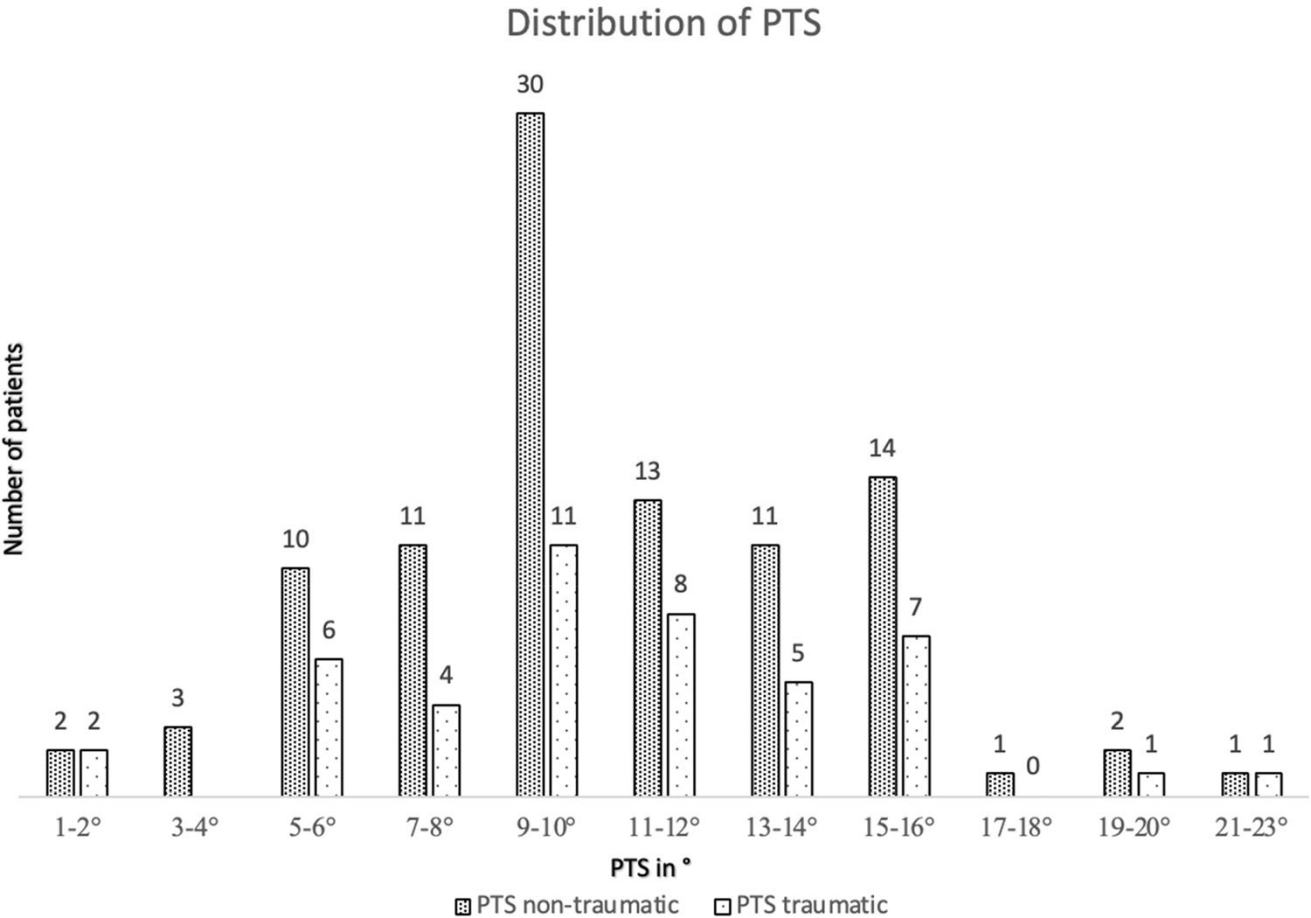
Distribution of PTS (°) values among patients with ACLR failure. ACLR, anterior cruciate ligament reconstruction; PTS, posterior tibial slope.

Patients underwent their most recent revision surgery at a mean interval of 58.5 ± 55.9 months following their previous ACL reconstruction. Surgical characteristics are presented in Table 2 and Figure 4. Surgical characteristics did not differ significantly between groups (*p* > 0.05).

**Table 2:** Surgical details of patients with ACL graft failure.

ACL graft fixation	
Tibial, *n* (%)	
Interference screw	124 (86.7%)
Suspensory button	19 (13.3%)
Femoral, *n* (%)	
Suspensory button	100 (69.9%)
Interference screw	43 (31.1%)
Graft type, *n* (%)	
Hamstring tendon	125 (87.4%)
Quadriceps tendon ipsilateral	11 (8.7%)
Patella tendon	7 (3.9%)
Concomitant surgeries, *N* (%)	
Medial meniscus surgery	69 (48.3%)
Lateral meniscus surgery	14 (9.8%)
Lateral extraarticular tenodesis	6 (4.2%)

Abbreviation: ACL, anterior cruciate ligament.

### Concomitant injuries

No significant differences were observed between the traumatic and non‐traumatic groups with respect to most concomitant injuries (*p* > 0.05). However, patients with non‐traumatic injury mechanisms demonstrated a significantly higher incidence of medial meniscus injuries compared to those with traumatic mechanisms (*n* = 53 [54.1%] vs. *n* = 16 [35.6%], *p* = 0.034) (Table 3). In traumatic cases, a semitendinosus graft was utilized in 38 patients (84.4%) and a quadriceps tendon graft in 7 patients (15.6%). Among traumatic cases, a semitendinosus graft was used in 84 patients (85.7%) and a quadriceps tendon graft in 14 patients (14.3%). Graft choice was not found to be statistically different within the groups (*p* > 0.05).

**Table 3:** Concomitant injuries in patients with ACL graft failure according to the mechanism of injury.

	Non‐traumatic	Traumatic	*p* value
Lateral meniscus injury (*n*)	22	16	0.11
Medial meniscus injury (*n*)	53	16	0.034
Medial cartilage defect (*n*)	24	5	0.06
Lateral cartilage defect (*n*)	12	2	0.12
Patellofemoral cartilage defect (*n*)	16	7	0.88

*Note*: Concomitant injuries and graft choice are presented as absolute values; *p* < 0.05 is considered significant.

Abbreviation: ACL, anterior cruciate ligament.

### Risk factor analysis

None of the analyzed variables demonstrated a statistically significant association (*p* > 0.05) with non‐traumatic ACLR failure (Table 4).

**Table 4:** Multivariate logistic regression of risk factors for non‐traumatic ACL retears.

Variable	Odds ratio	*p* value	95% CI
Age	1.03	0.13	0.99–1.08
Sex	0.65	0.29	0.29–1.44
Tibial tunnel	1.45	0.34	0.68–3.09
Femoral tunnel	0.77	0.53	0.36–1.71
ACL history	1.67	0.32	0.61–4.60
PTS	1.04	0.42	0.94–1.15
Femoral button fixation	2.25	0.07	0.14–1.56
Femoral screw fixation	0.43	0.06	0.18–1.05
Tibial button fixation	0.48	0.23	0.14–1.60
Tibial screw fixation	2.02	0.26	0.59–6.87

*Note*: Odds ratios are presented with corresponding 95% CIs, indicating the precision of the estimated effect sizes. A *p* value below 0.05 was considered statistically significant.

Abbreviations: ACL, anterior cruciate ligament; CIs, confidence intervals; PTS, posterior tibial slope.

### Interrater reliability

Interrater reliability was 0.76 (CI 0.54–0.89) for the measurement of PTS. Agreement for the femoral tunnel position was 90% (*k* = 0.79).

## DISCUSSION

The most important finding of this study was that PTS and non‐anatomic tunnel positioning were not associated with a non‐traumatic mechanism of ACLR failure. Furthermore, patient age, sex and graft fixation type demonstrated no significant influence on the occurrence of non‐traumatic ACLR failures. However, a higher prevalence of medial meniscus lesions was observed in the non‐traumatic cohort.

A higher PTS has frequently been discussed as a morphological risk factor for ACLR failure. While there is a correlation between increasing PTS and a higher ACL rerupture rate, several studies have demonstrated that a PTS exceeding 12° significantly increases anterior tibial translation, thereby imposing greater mechanical stress on the ACL graft [14, 15] and consequently increased the graft failure rate [13, 21, 30, 32]. However, it remains unclear if a higher PTS predisposes to non‐traumatic ACLR failure. In the present study, no statistically significant difference in PTS was observed between the traumatic and non‐traumatic failure groups. In contrast, previous research has demonstrated that patients with non‐traumatic ACLR failure exhibited a significantly higher medial and lateral PTS compared with those in a non‐failed ACLR cohort measured on MRI [22].

Besides PTS, non‐anatomic tunnel malpositioning, especially on the femoral side, is widely recognized as a major contributor to ACLR failure [5, 18, 22, 28]. Several studies have demonstrated that non‐traumatic failure is strongly associated with non‐anatomical femoral tunnel positioning [5, 22]. Anteriorly and vertically placed femoral tunnels, as well as laterally positioned tibial tunnels, are reported to predispose patients to non‐traumatic ACLR failure [22]. Similarly, femoral tunnel malposition—particularly when the tunnel is placed too anteriorly and proximally—has been associated with a higher rate of non‐traumatic ACLR failure compared to traumatic cases [5].

Nevertheless, the differing results of the aforementioned studies compared to the present findings are likely attributable to methodological variations. Whereas the previous studies compared absolute tunnel positions between failed and non‐failed reconstructions, the present investigation classified tunnel placement as either anatomic or extra‐anatomic based on predefined ranges corresponding to the native ACL insertion site [5, 22]. The aim of the present study was to determine whether non‐traumatic ACLR failures were more frequently associated with extra‐anatomic tunnel placement rather than to directly compare tunnel position measurements.

Another notable finding of our study is the significantly higher incidence of medial meniscus injuries in the non‐traumatic retear group. This may result from persistent microinstability or increased anteroposterior translation, leading to progressive medial meniscal degeneration and meniscal tears eventually. This observation aligns with biomechanical data demonstrating that, in ACL‐deficient knees, shear forces on the medial meniscus can double in comparison to ACL‐intact knees [19]. The medial meniscus is widely regarded as an important secondary stabilizer of the knee and is particularly vulnerable in cases of delayed ACL reconstruction or longstanding ACL insufficiency [29]. Previous studies have reported medial meniscus tear rates of 33.1% within the first 3 months after ACL injury, rising to 59.6% at 12 months post‐injury [11]. These findings underscore the clinical relevance of chronic microinstability, which may compromise meniscal integrity and predispose to non‐traumatic graft failure.

## LIMITATIONS

First, this is a retrospective design, which carries inherent limitations. Secondly, given the likely underpowered nature of the statistical model due to group size differences, subtle biomechanical effects may not have been detected. To add, multivariate regression can only capture linear relationships and may therefore overlook more complex or nonlinear patterns in the data. Patient‐reported outcome measures or clinical outcome data were not part of the research question and therefore not included. Additionally, pre‐existing medial meniscus injuries were not considered in the analysis, which may have influenced the observed higher prevalence of medial meniscus damage in the non‐traumatic group. Another limitation lies in the definition of the anatomical range for tunnel placement. While the defined anatomical placement is within ±2.5% of the native ACL insertion site, in the present study, a broader threshold of ±10% was applied, which appears clinically applicable.

Further, the results may also be biased by the large number of extra‐anatomic femoral tunnels in both groups. Also, retrospectively asking about the mechanism of trauma may introduce recall bias, and patients may underreport minor injuries, making it difficult to clearly distinguish between traumatic and non‐traumatic cases. Furthermore, most patients were referred from external institutions, which may have increased heterogeneity in surgical techniques.

## CONCLUSION

Patients in our cohort with non‐traumatic ACLR failure did not demonstrate a higher PTS or a higher incidence of non‐anatomically placed tibial or femoral tunnels compared with patients experiencing traumatic retears. However, non‐traumatic cases were associated with a higher prevalence of medial meniscus tears.

## AUTHOR CONTRIBUTIONS

Lorenz Fritsch, Lukas Willinger and Armin Runer designed the study. Luca Bausch and Lorenz Fritsch collected the data. Lorenz Fritsch and Romed P. Vieieder performed the statistical analysis. Lorenz Fritsch, Svenja Höger and Romed P. Vieieder wrote the manuscript. Lukas Willinger, Julian Mehl and Sebastian Siebenlist supported data interpretation and critically reviewed the manuscript. All authors read and approved the final manuscript.

## CONFLICT OF INTEREST STATEMENT

Sebastian Siebenlist is a consultant for Arthrex GmbH, KLS Martin Group and medi GmbH & Co. KG. Julian Mehl is a consultant for Arthrex GmbH and Ormed GmbH. The remaining authors declare no conflict of interest.

## ETHICS STATEMENT

The study was approved by the institutional review board of the Technical University of Munich (reference: 2023‐296‐S‐SR). Written informed consent was obtained from all patients.

## Data Availability

Data are available upon request.
